# Reduction of autophagy and increase in apoptosis correlates with a favorable clinical outcome in patients with rheumatoid arthritis treated with anti-TNF drugs

**DOI:** 10.1186/s13075-019-1818-x

**Published:** 2019-01-29

**Authors:** M. Vomero, V. Manganelli, C. Barbati, T. Colasanti, A. Capozzi, A. Finucci, F. R. Spinelli, F. Ceccarelli, C. Perricone, S. Truglia, S. Morrone, R. Maggio, R. Misasi, M. Bombardieri, M. Di Franco, F. Conti, M. Sorice, G. Valesini, C. Alessandri

**Affiliations:** 1 Arthritis Center, Department of Internal Medicine and Medical Specialties, Sapienza University of Rome, Rome, Italy; 2 Department of Experimental Medicine, Sapienza University of Rome, Rome, Italy; 3 Centre for Experimental Medicine and Rheumatology, William Harvey Research Institute, Queen Mary University of London, London, UK

**Keywords:** Autophagy, Apoptosis, Rheumatoid arthritis, TNFα, TNF inhibitors

## Abstract

**Background:**

Autophagy has emerged as a key mechanism in the survival and function of T and B lymphocytes, and its activation was involved in apoptosis resistance in rheumatoid arthritis (RA). To investigate whether the relationship between autophagy and apoptosis may impact the response to the therapy, we analyzed ex vivo spontaneous autophagy and apoptosis in patients with RA subjected to treatment with anti-tumor necrosis factor (TNF) drugs and in vitro the effects of TNFα and anti-TNF drugs on cell fate.

**Methods:**

Peripheral blood mononuclear cells (PBMCs) from 25 RA patients treated with anti-TNF drugs were analyzed for levels of autophagy marker LC3-II by western blot and for the percentage of annexin V-positive apoptotic cells by flow cytometry. The same techniques were used to assess autophagy and apoptosis after in vitro treatment with TNFα and etanercept in both PBMCs and fibroblast-like synoviocytes (FLS) from patients with RA.

**Results:**

PBMCs from patients with RA responsive to treatment showed a significant reduction in LC3-II levels, associated with an increased apoptotic activation after 4 months of therapy with anti-TNF drugs. Additionally, the expression of LC3-II correlated with DAS28. TNFα was able to induce autophagy in a dose-dependent manner after 24 h of culture in RA PBMCs and FLS. Moreover, etanercept caused a significant reduction of autophagy and of levels of citrullinated proteins.

**Conclusions:**

Our results show how the crosstalk between autophagy and apoptosis can sustain the survival of immune cells, thus influencing RA progression. This suggests that inhibition of autophagy represents a possible therapeutic target in RA.

**Electronic supplementary material:**

The online version of this article (10.1186/s13075-019-1818-x) contains supplementary material, which is available to authorized users.

## Background

Rheumatoid arthritis (RA) is a chronic autoimmune disease characterized by autoantibodies production as a result of breakage of immunological tolerance mechanisms [1]. Different immune cell types including fibroblast-like synoviocytes (FLS) and lymphocytes take part in both joints-restricted and extra-articular manifestations of the disease, and tumor necrosis factor alpha (TNFα) is clearly the most important cytokine in RA [2, 3]. Despite the relatively high efficacy of TNF antagonists, approximately one third of patients are intolerant or non-responsive to such drugs, thus the discovery of new therapeutic targets is desirable in RA management.

Autophagy (or macroautophagy) is an evolutionarily conserved lysosomal degradation pathway in eukaryotic organisms. During autophagy, portions of the cytoplasm are sequestered within characteristic double-membrane vesicles known as autophagosomes and are finally delivered to lysosomes for bulk degradation [4]. The products of autophagy-related gene (Atg) genes orchestrate every autophagy step ensuring the correct advancing of the process. Moreover, the levels of phosphatidylethanolamine-conjugated form of microtubule-associated protein light chain 3 (LC3)-II (LC3-II) are widely used to monitor autophagic activity in cells [5, 6]. Recently, aberrant autophagy regulation has been linked to a wide range of pathological conditions, including autoimmune diseases. In T lymphocytes from systemic lupus erythematosus (SLE) patients, an autophagy-resistant behavior has been demonstrated, and a critical role of this process is emerging in different aspects of RA pathogenesis [7, 8].

Autophagy actively participates in protein citrullination and carbamylation by substrates for the formation of autoantibodies [9, 10]. In fact, stimuli that induce autophagy were able to activate PAD4 enzyme in FLS from RA patients [9]. Also, ex vivo analysis in monocytes from early-naïve RA patients revealed an interesting direct correlation between autophagy levels and anti-cyclic citrullinated peptide (anti-CCP) titer. Recently, the same experimental condition was used to demonstrate that also carbamylation process was related to autophagy activation [10].

Increased autophagy levels were detected in synovial fibroblasts as well as in CD4+ T cells from RA patients, and a possible protective role of autophagy against apoptosis was suggested [11, 12]. Up to now, few studies have investigated the effect of TNF inhibitors on apoptosis with controversial results and, despite the importance of autophagy in immune-mediated mechanisms, how these drugs influence autophagy has not been clarified yet [13, 14].

In this study, to elucidate the possible role of autophagy in response to anti-TNF therapy, we analyzed the effect of anti-TNF drugs on the interplay between autophagy and apoptosis both ex vivo and in vitro in peripheral immune cells and synoviocytes from patients with RA.

## Methods

### Patients

Twenty-five consecutive RA patients, fulfilling the 2010 ACR/EULAR criteria, were recruited from the Rheumatology Unit of Sapienza University of Rome after signing a written informed consent [15]. The study was approved by the Ethics Committee of Sapienza University of Rome (protocol number 110/18). All patients were naïve for anti-TNF drugs and were followed after 4 months of treatment with these drugs. At every visit, clinical and laboratory assessments were performed and EULAR response criteria were used to classify participants as responder or non-responders to the therapy. For each patient, the following variables were recorded: clinical data included the disease duration and activity of RA, tender and swollen joint counts, and the patient’s general assessment of his/her condition scored on a visual analog scale (VAS). The disease activity of RA was determined in all patients with the Disease Activity Score on 28 joints (DAS28) using C-reactive protein and clinical disease activity index (CDAI).

### PMBCs and FLS isolation

Peripheral blood mononuclear cells (PBMCs) were isolated from RA patients by Ficoll–Hypaque density-gradient centrifugation. Layer of mononuclear cells was collected and then washed twice in phosphate-buffered saline (PBS) and cultured in RPMI 1640 medium with 10% FBS supplemented with 2 mM glutamine and 50 mg/ml gentamycin.

Synovial tissue was collected from patients with RA undergoing total knee replacement in London, after obtaining their informed consent. Ethical approval was granted by the East London and The City Research Ethics Committee 3 (LREC07/Q0605/29). The patients’ mean age was 73.25 years.

Fibroblast-like synoviocytes (FLS) were isolated immediately after surgery by digestion of the synovial tissue with Dispase at 37 °C for 60 min [16]. After washing, the cells were grown in a Dulbecco’s modification of Eagle medium (DMEM) supplemented with 10% FBS, 50 IU/ml penicillin/streptomycin, 2 mM glutamine, and 10 mM HEPES. For the experiments, synovial fibroblasts were used between passages 4 and 8. Primary human fibroblast cultures obtained from skin biopsy were used for preliminary experiments.

### Cell cultures

PBMCs and FLS were treated with recombinant human TNFα (R&D) at concentrations of 5 and 10 ng/ml for 24 h (time and concentrations were chosen after preliminary experiments) at 37 °C in a humidified incubator in an atmosphere of 5% CO2.

To induce autophagy, starvation was performed by incubating cells at lower concentration of fetal bovine serum (1% FBS). Autophagic flux was assessed by adding lysosomal protease inhibitors E64d and pepstatin A (Pep A, both at 10 mg/ml; Sigma) for 2 h before the end of the culture. For inhibition of autophagy, PBMCs were treated with 10 mM 3-methyladenine (3-MA; Sigma). Etanercept, purchased from the hospital pharmacy, was diluted in cell medium at concentration of 15 μg/mL. PBMCs and FLS were maintained in the incubator at 5% CO2 and 37 °C and after 24 h of culture were collected for analysis of autophagy, apoptosis, and citrullination.

### Apoptosis detection

Apoptosis was measured using FITC-conjugated annexin V (AV) and a propidium iodide (PI) apoptosis detection kit (Marine Biological Laboratory) following manufacturer’s protocol. Fifty thousand events for each sample were run on a FACSCalibur cytometer, and data were analyzed using the CellQuest Pro software (BD). Reported data refer to AV-positive apoptotic cells.

### Cell sorting

For the separation of CD4+ T, CD8+ T, and B lymphocytes, PBMCs from patients with RA were stained with surface antigen-specific antibodies (Additional file 1: Figure S1) and cell sorting was performed with the flow cytometer FACS Aria (BD Biosciences). Purity of the enriched populations was greater than 99% in all experiments.

### Western blot

RA FLS and PBMCs were lysed in RIPA buffer in the presence of complete protease-inhibitor mixture (Roche). Lysates (50 μg) were loaded on 15% (for autophagy analysis), to 10% (for citrullinated proteins detection) and to 7.5% (for PARP detection) SDS-polyacrylamide gel, and then electrophoretically transferred onto PVDF membranes. The membranes were incubated overnight at + 4 °C shaking with rabbit anti-human LC3B antibody (Cell Signaling Technology), rabbit anti-human PARP antibody (Cell Signaling Technology), rabbit anti-human β-actin antibody (Sigma), and rabbit polyclonal anti-citrulline antibody (Millipore). The immunoreactivity was assessed by a chemiluminescence reaction, and densitometry analysis was used for the quantification of protein expression on autoradiograms.

### Anti-CCP antibodies assay

Quantification of serum levels of anti-CCP antibodies was determined by commercial ELISA kit (Delta Biologicals) in accordance with the manufacturer’s instructions. A value of anti-CCP antibodies ≥ 30 U/mL was considered positive.

### Statistical analysis

Data are expressed as mean ± standard deviation (SD) or median (interquartile range). Differences between groups were analyzed using the Mann-Whitney *U* test, and Spearman test was used for correlation analysis. To analyze the changes in autophagy and apoptosis levels after therapy, the Wilcoxon signed rank test was used. *P* values <0.05 were considered statistically significant.

## Results

### Clinical and serological characteristics of RA patients

Twenty-five patients with established RA naïve to biological agents (23 females and 2 males, mean age 59 years, mean duration of disease 6.3 years) were included in this study. The baseline demographic, clinical, and laboratory parameters are shown in Table 1. In our cohort, 72% of patients with RA were positive for anti-CCP antibodies and at time zero no clinical differences were observed between anti-CCP positive and negative patients. An additional number of eight patients with RA were enrolled for sorting experiments (Additional file 1: Table S1). After the failure of conventional synthetic disease-modifying anti-rheumatic drug (csDMARDs), all the patients started therapy with anti-TNF agents [20 patients received etanercept (50 mg/week) and 5 adalimumab (40 mg/2 weeks)]. Thirteen patients were in treatment with anti-TNF drugs plus methotrexate (MTX, 10–20 mg weekly).

**Table 1:** Baseline clinical and serological characteristics of patients with RA

Characteristic	Value
Demographic parameters	
Sex, F/M	23/2
Age, mean (SD), years	59 (12.6)
Disease duration, mean (SD), years	6.3 (8.8)
Laboratory parameters	
ESR, mean (mm/h) (SD)	17.6 (9.3)
CRP, mean (mg/dL) (SD)	0.68 (0.65)
RF positivity, n (%)	19 (76)
ACPA positivity, n (%)	18 (72)
Disease activity	
TJ n, mean (SD)	5.8 (5.4)
SJ n, mean (SD)	2.6 (2.4)
CDAI, mean (SD)	19.3 (10.8)
DAS28, mean (SD)	4.2 (1.6)
Therapy	
Etanercept, n (%)	20 (80)
Adalimumab, n (%)	5 (20)
Concurrent MTX, n (%)	13 (52)

*SD* standard deviation, *ESR* Erythrocyte Sedimentation Rate, *CRP* C-Reactive Protein, *RF* Rheumatoid Factor, *ACPA* anti-citrullinated peptide antibodies, *TJ* Tender joints, *SJ* swollen joints, *CDAI* Clinical Disease Activity Index, *DAS28* Disease Activity Score on 28 joints, *csDMARDs* conventional synthetic disease-modifying antirheumatic drugs

### Spontaneous autophagy and apoptosis in RA patients before and after treatment with anti-TNF drugs

To evaluate a possible relationship between autophagy and RA progression, we analyzed the levels of spontaneous autophagy at baseline (t0) and after 4 months of treatment (t4) with anti-TNF drugs in PBMCs isolated from patients with RA. Patients were divided into two groups according to the clinical response: we merged good and moderate responders against non-responders. As expected, the treatment significantly reduced DAS28 score in patients responding to treatment (from 4.3 ± 1.5 to 2.5 ± 1.1, *P* = 0.002). Conversely, no significant clinical improvement was found in non-responders at 4 months of follow-up.

**Fig. 1:**
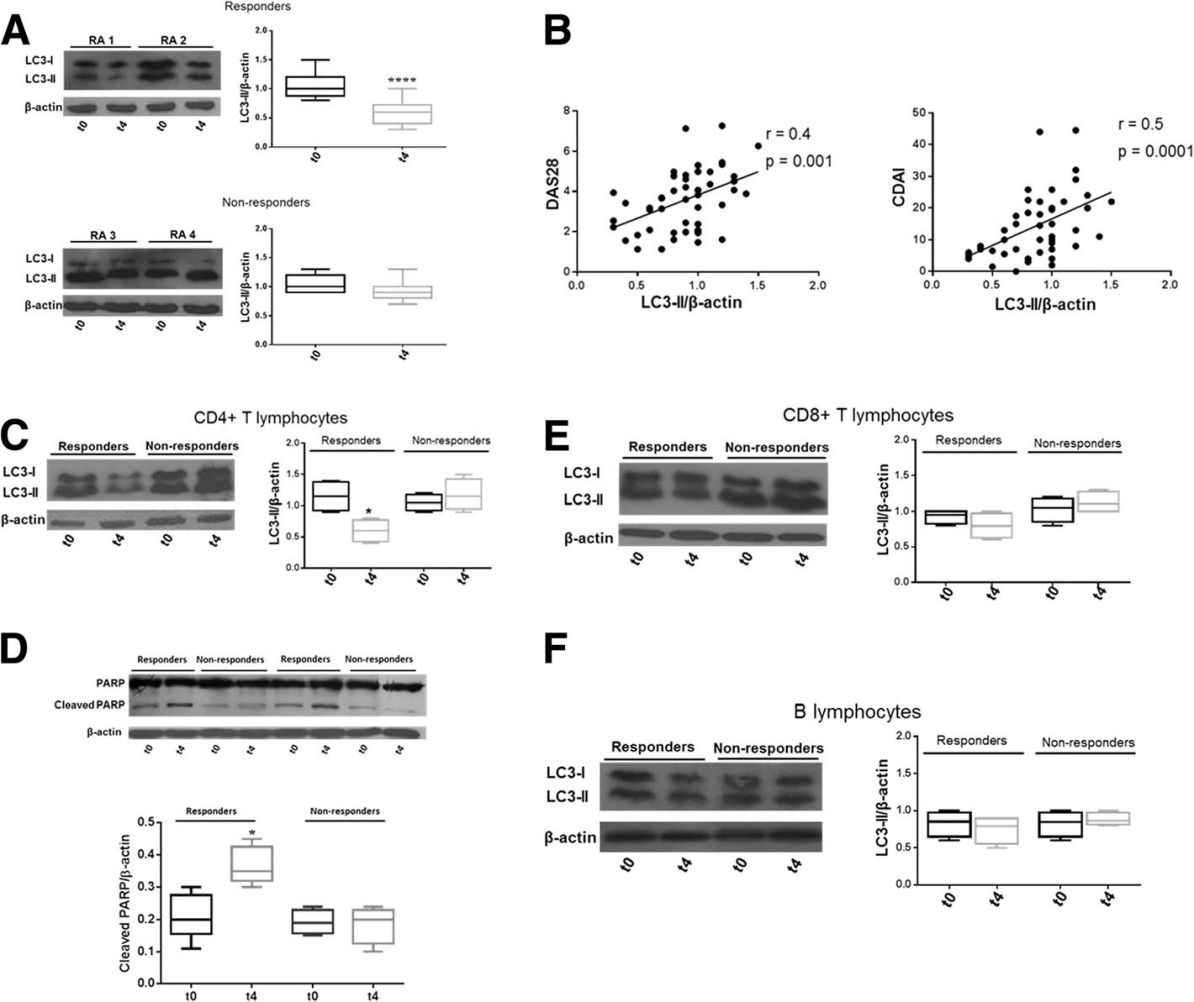
Autophagy levels in PBMCs from RA responders and non-responders to anti-TNF drugs. **a** Western blot analysis of LC3-II in PBMCs isolated from patients with RA before (t0) and after 4 months of treatment with anti-TNF drugs (t4). Samples from two responding and two non-responding patients as representative of the total number of 25 analyzed are shown. Densitometry analysis of LC3-II levels relative to β-actin is also displayed. ****P < 0.0001. **b** Correlation between autophagy and clinical parameters in PBMCs isolated from patients with RA at baseline and after therapy with anti-TNF drugs. Spearman’s rank correlation coefficient and linear regression are displayed. DAS 28, Disease Activity Score 28; CDAI, clinical disease activity index. **c**, **d** Autophagy and apoptosis levels in CD4+ T lymphocytes from responders and non-responders. Western blot analyses of LC3-II (**c**) and of cleaved PARP (**d**) in CD4+ T lymphocytes from patients with RA. Densitometry analyses are also shown. *P < 0.05. **e**, **f** Autophagy levels in CD8 + T and B lymphocytes from responding and non-responding patients before and after treatment with anti-TNF drugs. Western blot analysis of autophagy marker LC3-II in cell lysates obtained from CD8+ (**e**) and CD19+ (**f**) lymphocytes purified by cell sorting. Blots shown are representative of independent experiments performed in T and B cells from eight patients with RA before and after treatment with anti-TNF drugs. Densitometry analysis of LC3-II amount relative to β-actin is also shown

We did not find differences in autophagy levels after treatment with TNF inhibitors (data not shown); however, when patients with RA were divided based on response to the therapy, we noticed that LC3-II levels significantly decreased in PBMCs from responders (*n* = 17, Fig. 1a). On the contrary, no significant change in autophagy was found in patients non-responding to the therapy (*n* = 8, Fig. 1a). In addition, a positive correlation between LC3-II levels and both DAS28 and CDAI was observed (Fig. 1b).

Since MTX was found to induce autophagy in RAFLS [17] and 13 patients of our cohort were taking anti-TNF drugs in association with MTX, we evaluated the results in light of this issue. As displayed in Additional file 1: Figure S1, the reduction of autophagy and the increase in apoptosis in responders was not influenced by concomitant MTX use.

The analysis of serum levels of anti-CCP antibodies in responding patients revealed no statistically significant reduction of anti-CCP titer after anti-TNF therapy. In addition, no correlation with LC3-II levels and with the percentage of AV-positive cells was found. In responders, autophagy and apoptosis were reduced both in patients that were positive and negative for anti-CCP antibodies (Additional file 1: Figure S2).

In order to clarify which lymphocyte subsets could be involved in autophagy-dependent response to therapy, we analyzed autophagy marker LC3-II in CD4+ and CD8+ T and B lymphocytes from patients with RA isolated by cell sorting technique (Additional file 1: Figure S3). After 4 months of treatment with TNF antagonists, CD4+ T lymphocytes from responders showed a statistically significant reduction of LC3-II levels compared to the baseline (Fig. 1c). No significant change in autophagy was found in CD8+ T cells and B cells from both responders and non-responders to the therapy (Fig. 1e, f). Moreover, the analysis of the cleaved form of PARP confirmed the increased levels of apoptosis after therapy in CD4+ T cells from responders (Fig. 1d).

**Fig. 2:**
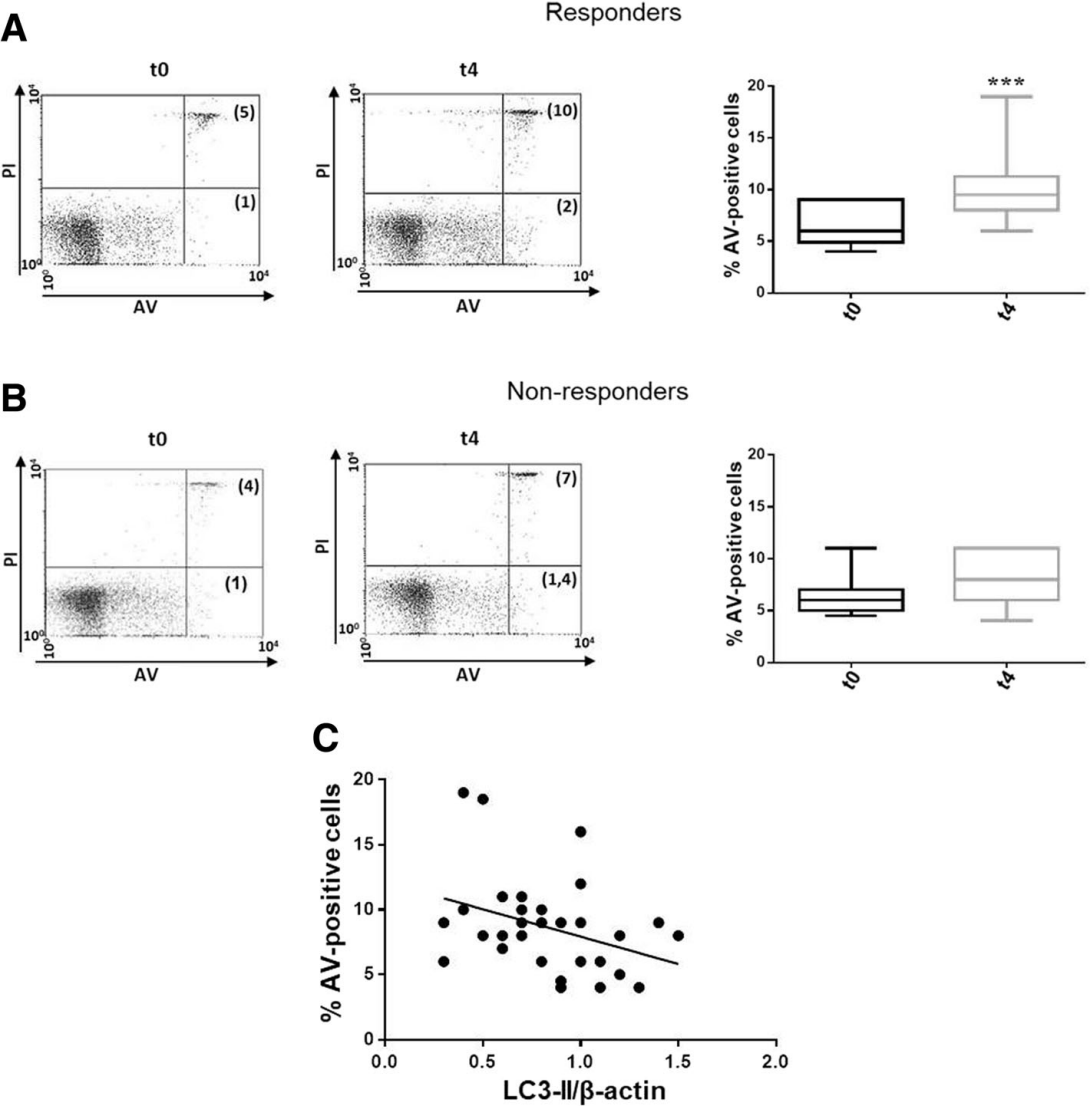
Analysis of spontaneous apoptosis in PBMCs purified from patients with RA treated with anti-TNF drugs. **a**, **b** Percentage of annexin V (AV)-positive cells by flow cytometry in PBMCs purified from patients with RA (*n* = 25) before (t0) and after 4 months (t4) of treatment with anti-TNF drugs. Analysis of apoptosis was performed in responders (*n* = 17) (**a**) and non-responders (*n* = 8) (**b**), separately. Representative dot plots (PI on *y*-axis vs. AV on *x*-axis) are also shown, ***P < 0.001. **c** Correlation and linear regression analysis of autophagy and apoptosis expressed as LC3-II levels and percentage of AV-positive cells, respectively, in PBMCs from patients with RA responding to anti-TNF therapy

Considering the crucial role of autophagy in the regulation of lymphocyte survival and the downregulation of apoptosis observed in immune cells of patients with RA, we focused on the variation in apoptosis in relation to response to anti-TNF therapy [18, 19]. In patients responding to anti-TNF therapy, the percentage of AV-positive apoptotic cells increased after 4 months of treatment (Fig. 2a), while the failure of the therapy was associated with unchanged spontaneous apoptosis levels (Fig. 2b). Moreover, a significant inverse correlation between LC3-II levels and the percentage of apoptotic cells was observed in PBMCs from responders (Fig. 2c).

### Effect of TNFα on autophagy in PBMCs from patients with RA

**Fig. 3:**
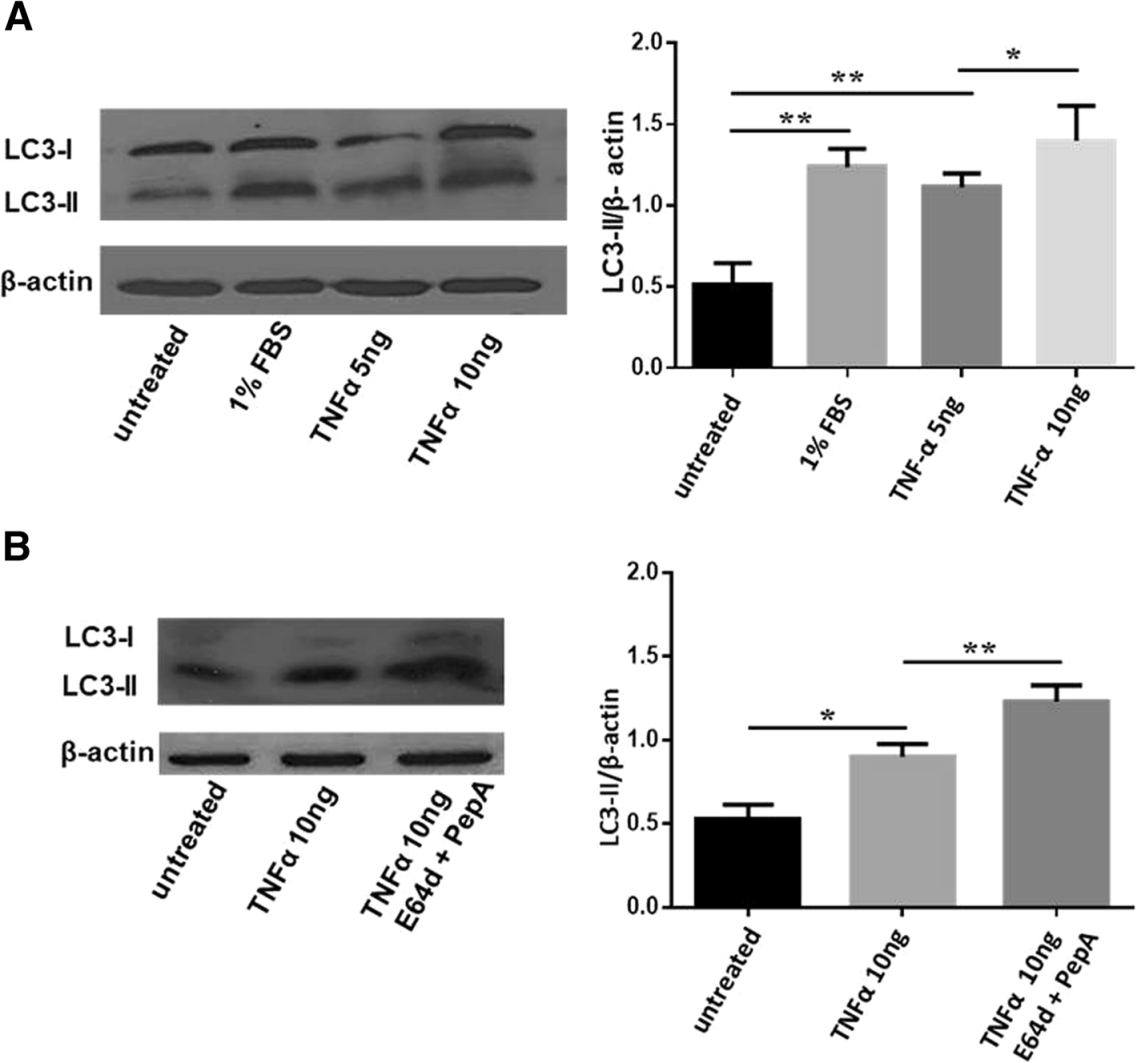
Effect of TNFα on autophagy in PBMCs from patients with RA. **a** Western blot analysis of LC3-II levels in PBMCs treated with TNFα at concentration of 5 and 10 ng/mL for 24 h. As a control condition, cells were cultured in serum deprivation condition (1% FBS). Blots shown are representative of five independent experiments performed in PBMCs from different patients with RA naïve to anti-TNF therapy. Densitometry analysis of LC3-II levels relative to β-actin is also shown. **P < 0.01, *P < 0.05. **b** Western blot and densitometry analysis of LC3-II levels in lysates from PBMCs treated with TNF-α and, when indicated, with E64d and PepA (added 2 h before the end of the culture). The figure was chosen as a representative of those obtained from five independent experiments on cells from different RA patients never treated with anti-TNF drugs. **P < 0.01

Several studies have demonstrated that TNFα was able to stimulate autophagy in different cell types, including RAFLS [20–22]. Here, we confirmed the pro-autophagic effect of this cytokine also in PBMCs from patients with RA in a dose-dependent manner (Fig. 3a). On the contrary, incubation with TNFα did not significantly affect apoptosis (Additional file 1: Figure S4). To assess whether the accumulation of LC3-II after stimulation with TNFα was due to upstream or downstream autophagy defect, the autophagic flux was assayed in presence of the lysosomal proteases inhibitors E64d and Pep A, two blockers of the degradation of the autophagolysosome content [6]. In the presence of these inhibitors, TNFα caused a further increase in LC3-II levels, confirming the ability of TNFα to induce autophagy in PBMCs from patients with RA (Fig. 3b).

**Fig. 4:**
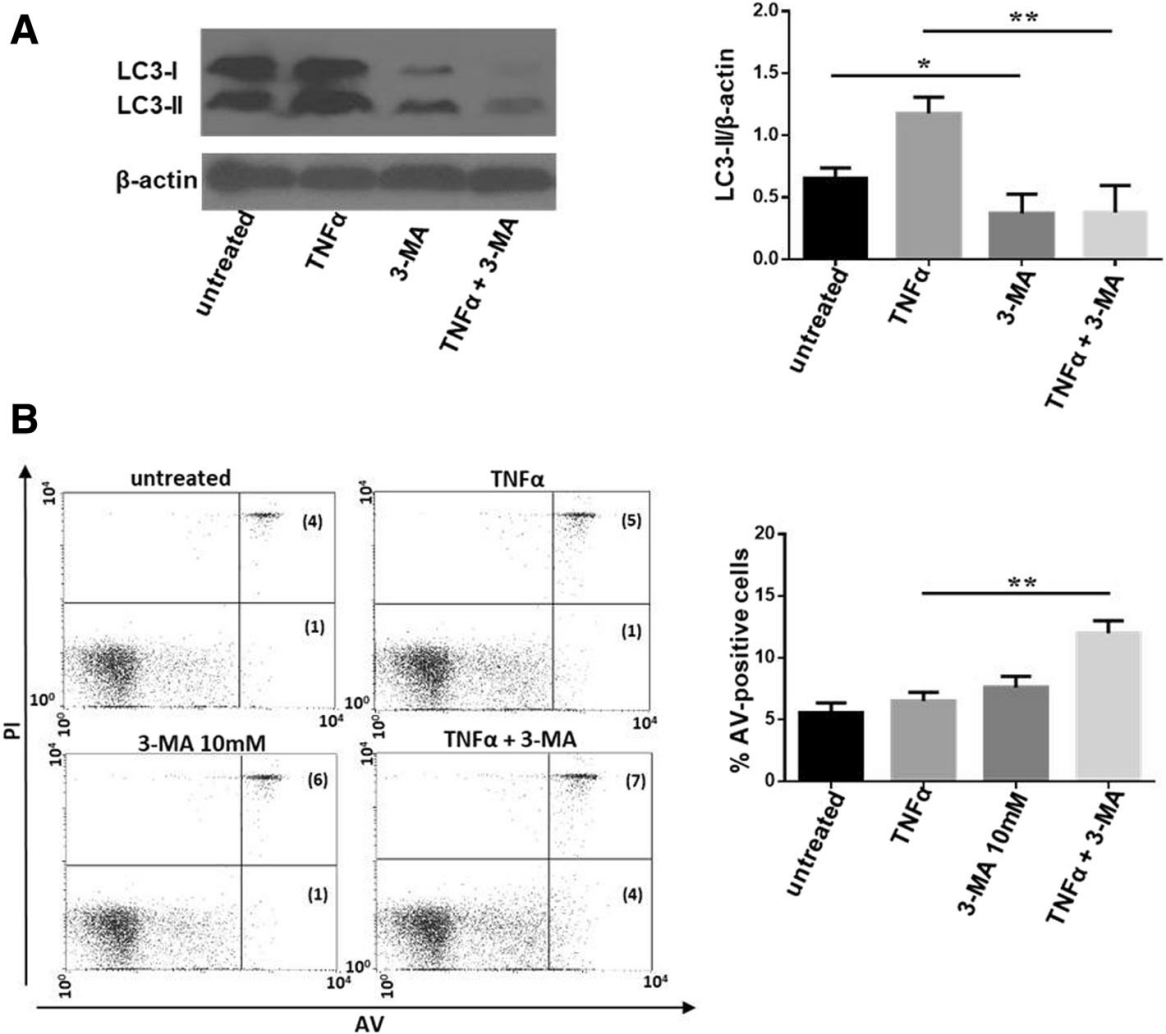
Effect of autophagy inhibition in PBMCs from patients with RA treated with TNFα. **a** Western blot analysis of LC3-II in PBMCs treated with the autophagy inhibitor 3-MA (10 mM) and TNFα (10 ng/mL) for 24 h. Blot Blot is representative representative of five independent experiments. Densitometry analysis of LC3-II relative to β-actin is also shown, **P < 0.01, *P < 0.05. **b** Statistical analysis of apoptosis of PBMCs isolated from patients with RA after treatment with 3-MA and TNFα. Results are expressed as AV-positive cells. Representative dot plots (PI on *y*-axis vs. AV on *x*-axis) are also shown, **P < 0.01

Considering the pro-autophagic effect of TNFα and the enhanced apoptosis in patients responding to anti-TNF therapy, we analyzed whether autophagy inhibition in cells treated with TNFα affected apoptosis in vitro*.* To this aim, PBMCs from patients with RA were treated with TNFα in association with the autophagy inhibitor 3-MA for 24 h. As expected, LC3-II levels were reduced after 3-MA treatment (Fig. 4a). Interestingly, the co-treatment with TNFα and 3-MA caused a significant increase in apoptosis (Fig. 4b), suggesting that autophagy induced by TNFα was able to protect RA PBMCs from apoptosis.

### Effect of etanercept on autophagy, apoptosis, and citrullination in PBMCs isolated from patients with RA

**Fig. 5:**
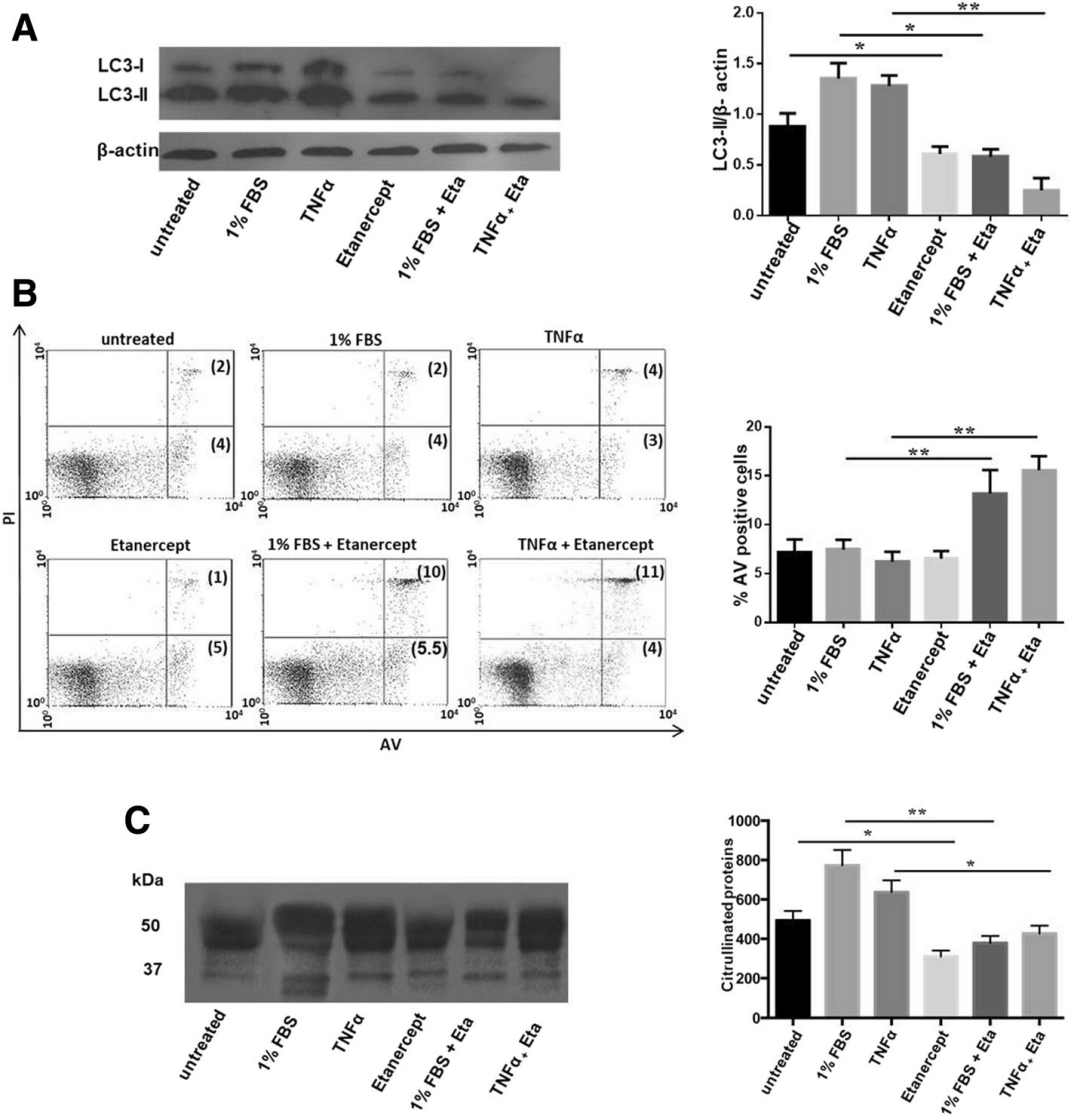
Evaluation of autophagy, apoptosis, and citrullination after in vitro treatment with etanercept in PBMCs from patients with RA. **a** LC3-II levels in PBMCs purified from patients with RA after in vitro treatment with etanercept at concentration of 15 μg/ml. Where indicated, cells were starved (1% FBS) or incubated with TNFα for 4 h, and then etanercept was added to the culture for 24 h. Densitometry analysis obtained in five independent experiments is also shown, **P < 0.01, *P < 0.05. **b** Flow cytometry analysis of apoptosis after in vitro treatment with etanercept as previously described. Results, referred to five independent experiments in PBMCs isolated from patients with RA, are expressed as percentage of AV-positive apoptotic cells. Representative dot plots are shown (PI on *y*-axis vs. AV on *x*-axis), **P < 0.01. **c** Levels of citrullinated proteins in PBMCs isolated from patients with RA treated with etanercept in vitro. Densitometric analysis is also shown. Data are obtained from five independent

In order to possibly reproduce the in vivo conditions, RA PBMCs were cultured in serum deprivation or in presence of TNFα for 4 h, and then TNF-inhibitor was added to the culture. After 24 h, autophagy, apoptosis, and citrullination were evaluated. We used PBMCs from RA patients naïve to anti-TNF therapy to avoid any influence of a previous exposition to anti-TNF on results. The treatment with etanercept caused a statistically significant reduction of LC3-II levels; moreover, inhibition of autophagy by etanercept resulted more marked when cells were exposed to TNFα and starvation (Fig. 5a). Etanercept alone did not affect the percentage of AV-positive cells, but interestingly a significant change in apoptosis was obtained only when this compound was added after pre-treatment with TNFα and in nutrient deprivation condition (Fig. 5b).

Recently, different studies evaluated the role of autophagy in the citrullination process, demonstrating that autophagy is directly involved in the generation of citrullinated proteins, both in synoviocytes and monocytes [9]. Here, in order to confirm the inhibitory effect of etanercept on autophagy, we analyzed levels of citrullinated proteins after in vitro treatment with TNFα and etanercept in PBMCs isolated from RA patients. Lysates from PBMCs analyzed by anti-citrulline antibody showed the presence of numerous bands corresponding to citrullinated proteins. Densitometric analysis revealed a statistically significant reduction reduction in the total amount of citrullinated proteins following incubation with etanercept, confirming a direct relation between autophagy and citrullination (Fig. 5c).

### Modulation of autophagy and apoptosis by etanercept in FLSs from RA patients

**Fig. 6:**
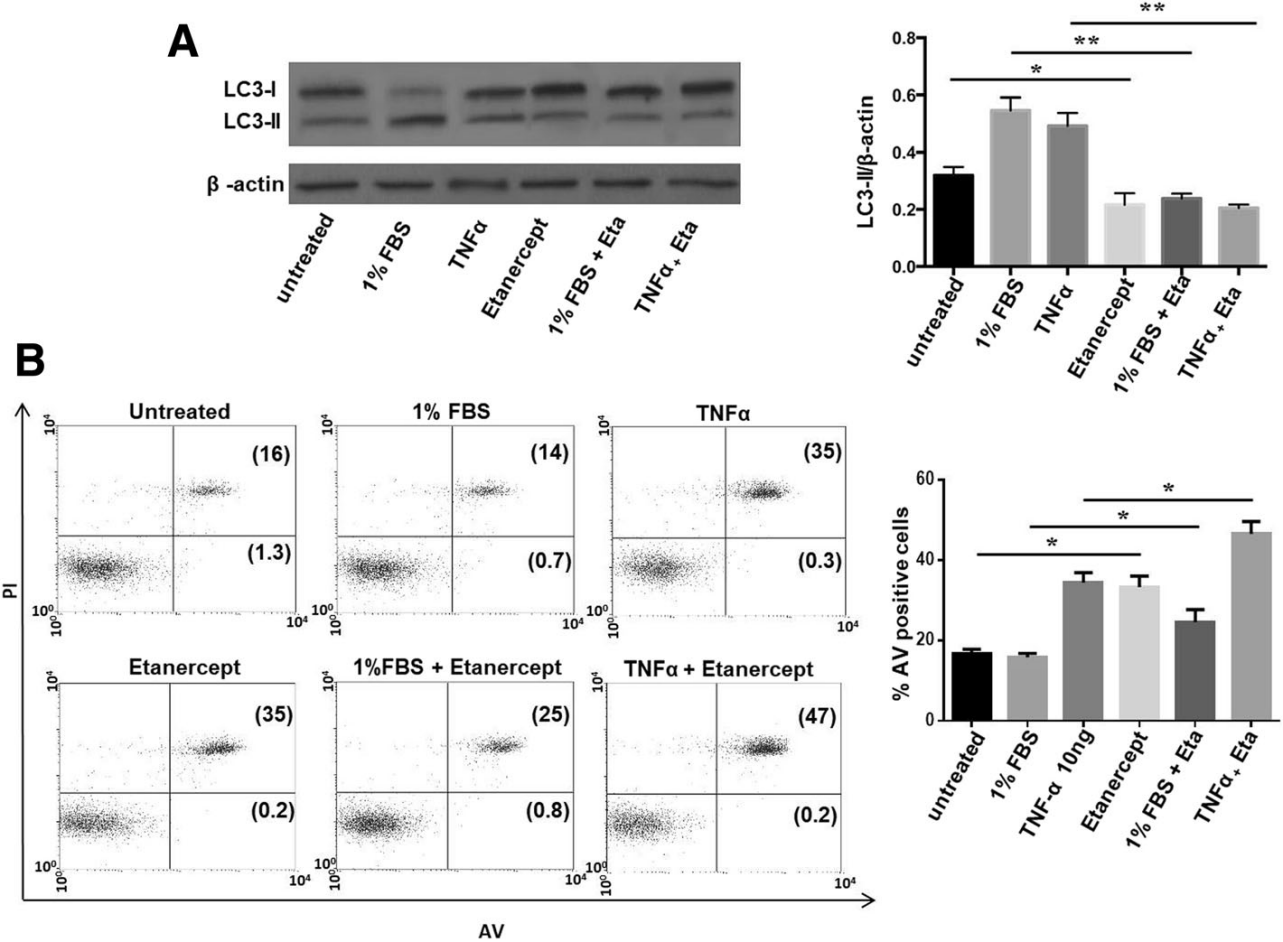
Autophagy and apoptosis levels after in vitro treatment with etanercept in FLS isolated from patients with RA. **a** Western blot analysis of LC3-II levels in FLS treated with etanercept at concentration of 15 μg/ml. Where indicated, cells were starved (1% FBS) or cultured in the presence of TNFα for 4 h and etanercept was then added for a total treatment duration of 24 h. Densitometry analysis of LC3-II expression obtained from five independent experiments is reported. **b** Flow cytometry analysis of apoptosis after in vitro treatment with etanercept as above described. Data, referred to five independent experiments, are expressed as percentage of AV-positive apoptotic cells. Representative dot plots are also shown (PI on *y*-axis vs. AV on *x*-axis), *P < 0.05

Since RA most significant pathogenic events occur in the synovium, we explored autophagic behaviour in response to in vitro treatment with etanercept also in FLSs isolated from patients with RA. Following preliminary experiments conducted on primary fibroblasts (data not shown), the dosage of etanercept chosen for the experiments was the same as the one used for PBMCs. Autophagy and apoptosis were increased after 24 h of treatment with TNFα in RA FLS (Fig. 6a). The adding of etanercept after treatment with TNFα and starvation caused a significant reduction in LC3-II levels as evidence of autophagy inhibition (Fig. 6a). In contrast to the data obtained in peripheral cells, etanercept was able to induce apoptosis in RA FLS. In addition, a further increase in AV-positive cells was found when the treatment with etanercept was performed in conditions that activate autophagy like starvation and treatment with TNFα (Fig. 6b).

## Discussion

In this study, we clarified the role of autophagy in the progression of RA by analyzing how the treatment with anti-TNF drugs modulated autophagy both ex vivo and in vitro. Although TNF inhibitors have revolutionized the clinical approach of inflammatory immune-mediated diseases, a substantial proportion of patients fails to respond to anti-TNF therapy, reducing therapeutic options and possibly leading to a rapid progression of the disease [23, 24]. For this reason, the study of mechanisms influencing the response to therapy has a crucial importance in the identification of new therapeutic targets in RA. Here, we analyzed the changes in spontaneous autophagy in peripheral cells from patients with RA treated with TNF inhibitors, demonstrating a reduction of autophagy only in patients responding to the therapy. As a result, the amount of LC3-II (at baseline and after anti-TNF therapy) directly correlated with disease activity score, showing that levels of autophagy may be a measure of disease progression. Our results in peripheral cells are in line with the data of Zhu and colleagues performed in the synovial tissue of active RA patients [25]. Given that B and T lymphocytes need autophagy for their maturation and activation [26], we extended our analysis in lymphocyte subsets, reporting a reduction of LC3-II levels and an increase in apoptosis also in CD4+ T lymphocytes from responding patients. Although these results must be confirmed in a large population, as already demonstrated in total peripheral immune cells, also in CD4+ T lymphocytes autophagy and apoptosis did not change in patients who failed to respond to the treatment. These experimental evidences suggest the possible use of autophagy markers as expression of disease activity and response to therapy in RA.

Autophagy is a physiological process that allows cells, including lymphocytes, to survive during stress conditions [27]. In this regard, recent papers deepened this aspect in the context of RA pathogenesis, suggesting a direct involvement of autophagy in apoptosis resistance in FLS and T cells [28]. Nonetheless, how the relationship between these two processes impacts the response to therapy has not been clarified yet. We demonstrated that the percentage of apoptotic cells increased in PBMCs from responding patients, and autophagy and apoptosis were found to be inversely correlated in this group of patients. Similar results were obtained by other researchers at synovial level [29]. Our findings suggest that autophagy participates in RA progression by promoting the survival of inflammatory and autoreactive cells, thus impacting the response to therapy. In fact, we hypothesize that PBMCs from patients with RA could take advantage of autophagy to survive and to supply energy for several pathological processes including migration to synovium.

By elimination of intracellular pathogens, delivered and degraded into autophagosomes, autophagy has a crucial role in the modulation of innate and adaptive immunity response. For this reason, it is not surprising that cytokines involved in cellular defense, such as TNFα, are also able to activate autophagy in different cell types [30]. Referring to the last guidelines for monitoring autophagy, we analyzed the autophagic flux in RA PBMCs treated with TNFα using lysosomal proteases inhibitors [6]. Blocking the degradation of autophagolysosome content, these molecules allow to understand if the increased levels of LC3-II after incubation with TNFα were due to an effective enhanced autophagy or rather to a defect on autophagosome-lysosome fusion [7]. The use of lysosomal proteases inhibitors increased autophagy levels in RA PBMCs treated with TNFα compared with cells treated with TNFα alone, confirming the ability of this cytokine to induce autophagy. Moreover, we found that treatment with TNFα in the presence of autophagy inhibitor 3-MA made RA PBMCs more susceptible to apoptosis induction. This result shows that autophagy contributes to apoptosis resistance in these cells. In addition, our results, according to previous published data in a collagen-induced arthritis (CIA) mouse model, suggest that the blockade of autophagy may be an interesting clinical approach in RA [31].

Patients enrolled in this study were treated with TNF inhibitors, drugs that suppress biological functions of TNFα. It was indirectly noticed that the reactivation of tuberculosis during anti-TNF therapy may be associated with autophagy suppression [32], but the effect of TNF inhibitors on autophagy has not been investigated yet in vitro. We found that exposition to etanercept, both alone and in conditions that stimulate autophagy (TNFα and nutrient deprivation), caused a significant reduction in LC3-II levels in cells from patients with RA.

Consistent with previous literature data [14], differences in ability of etanercept to induce apoptosis between synoviocytes and peripheral cells were found also in this study. While in FLS apoptosis was increased after treatment with etanercept; in PBMCs from patients with RA, a significant variation in apoptosis was found only in the presence of TNFα and nutrient deprivation.

Since citrullination may be considered an additional marker of autophagy in RA [33, 34], we analyzed the levels of citrullinated proteins in PBMCs from patients with RA after incubation with etanercept. According to our previous paper [9], stimuli that induce autophagy caused also an increase of citrullination. Furthermore, etanercept reduced levels of citrullinated proteins, confirming the inhibitory effect of etanercept on autophagy.

## Conclusions

To our knowledge, this is the first demonstration of a direct involvement of the interplay between autophagy and apoptosis in the response to therapy in patients with RA. Although functions of autophagy appear to be tissue and time specific, taken together, our data show how autophagy activation can sustain survival of immune cells from RA patients, influencing the effectiveness of the therapy. In fact, the restoration of apoptosis mediated by suppression of TNF-mediated autophagy may be another mechanism of action to explain the therapeutic effect of etanercept. Further studies in a large cohort of patients are needed to clarify whether a clinical approach based on autophagy inhibition may be beneficial in RA.

## Additional file

Additional file 1: **Figure S1.** Autophagy and apoptosis levels in PBMCs isolated from responding (A) and non-responding (B) patients to anti-TNF drugs alone or anti-TNF drugs plus methotrexate. **Figure S2.** Relation between autophagy and citrullination in RA patients. A. Levels of anti-cyclic citrullinated peptide antibodies (anti-CCP Abs) in patients with RA before and after treatment with anti-TNF drugs. Values are expressed as means ± sd. B. Correlation between anti-CCP antibodies levels (U/ml) and spontaneous autophagy (expressed as LC3-II) and apoptosis (expressed as percentage of Annexin V-positive cells). C. Changes in autophagy and apoptosis before and after treatment with anti-TNF drugs in relation to anti-CCP in RA responding patients. **Figure S3.** Flow cytometry gate strategy for purification of CD4+, CD8+ T lymphocytes and B lymphocytes from patients with RA. Allophycocyanin = APC; Peridinin chlorophyll protein = PerCP; Phycoerythrin = PE; Fluorescein isothiocyanate = FITC. **Figure S4.** Effect of TNFα on apoptosis. Flow cytometry analysis of apoptosis in PBMCs after treatment with TNFα. Apoptosis is expressed as percentage of AV-positive cells. Representative dot plots (PI on y axis vs. AV on x axis), chosen as representative of five experiments, are also shown. **Table S1.** Clinical, demographic and serological characteristics of patients with RA enrolled for sorting experiments (*n* = 8). (PDF 673 kb)

## Abbreviations

3-MA: 3-Methyladenine
Anti-CCP: Anti-cyclic citrullinated peptide
AV: Annexin V
CDAI: Clinical disease activity index
DAS28: Disease Activity Score on 28 joints
FBS: Fetal bovine serum
FLS: Fibroblast-like synoviocytes
LC3: Microtubule-associated protein light chain 3
MTX: Methotrexate
PBMCs: Peripheral blood mononuclear cells
Pep A: Pepstatin A
PI: Propidium iodide
RA: Rheumatoid arthritis
SD: Standard deviation
TNFα: Tumor necrosis factor alpha
