# Supplementary material for: Reduction of autophagy and increase in apoptosis correlates with a favorable clinical outcome in patients with rheumatoid arthritis treated with anti-TNF drugs
Source: Arthritis Res Ther. 2019 Jan 29;21:39. doi: 10.1186/s13075-019-1818-x (PMC6352385; doi:10.1186/s13075-019-1818-x)
Supplement: Supplementary file 1 — Figure S1. Autophagy and apoptosis levels in PBMCs isolated from responding (A) and non-responding (B) patients to anti-TNF drugs alone or anti-TNF drugs plus methotrexate. Figure S2. Relation between autophagy and citrullination in RA patients. A. Levels of anti-cyclic citrullinated peptide antibodies (anti-CCP Abs) in patients with RA before and after treatment with anti-TNF drugs. Values are expressed as means ± sd. B. Correlation between anti-CCP antibodies levels (U/ml) and spontaneous autophagy (expressed as LC3-II) and apoptosis (expressed as percentage of Annexin V-positive cells). C. Changes in autophagy and apoptosis before and after treatment with anti-TNF drugs in relation to anti-CCP in RA responding patients. Figure S3. Flow cytometry gate strategy for purification of CD4+, CD8+ T lymphocytes and B lymphocytes from patients with RA. Allophycocyanin = APC; Peridinin chlorophyll protein = PerCP; Phycoerythrin = PE; Fluorescein isothiocyanate = FITC. Figure S4. Effect of TNFα on apoptosis. Flow cytometry analysis of apoptosis in PBMCs after treatment with TNFα. Apoptosis is expressed as percentage of AV-positive cells. Representative dot plots (PI on y axis vs. AV on x axis), chosen as representative of five experiments, are also shown. Table S1. Clinical, demographic and serological characteristics of patients with RA enrolled for sorting experiments (n = 8). (PDF 673 kb) [file 13075_2019_1818_MOESM1_ESM.pdf]

Supplementary Material

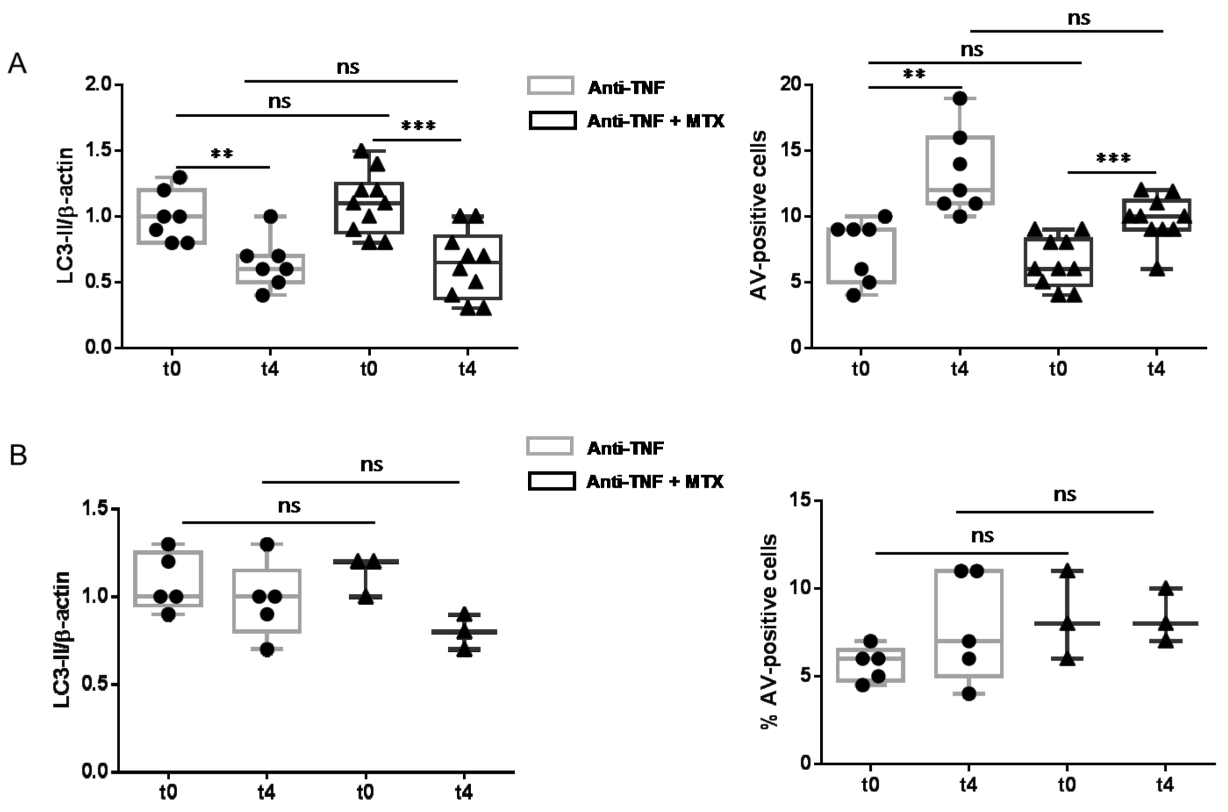

Supplementary Figure 1. Autophagy and apoptosis levels in PBMCs isolated from responding (A) and non-responding (B) patients to anti-TNF drugs alone or anti-TNF drugs plus methotrexate.

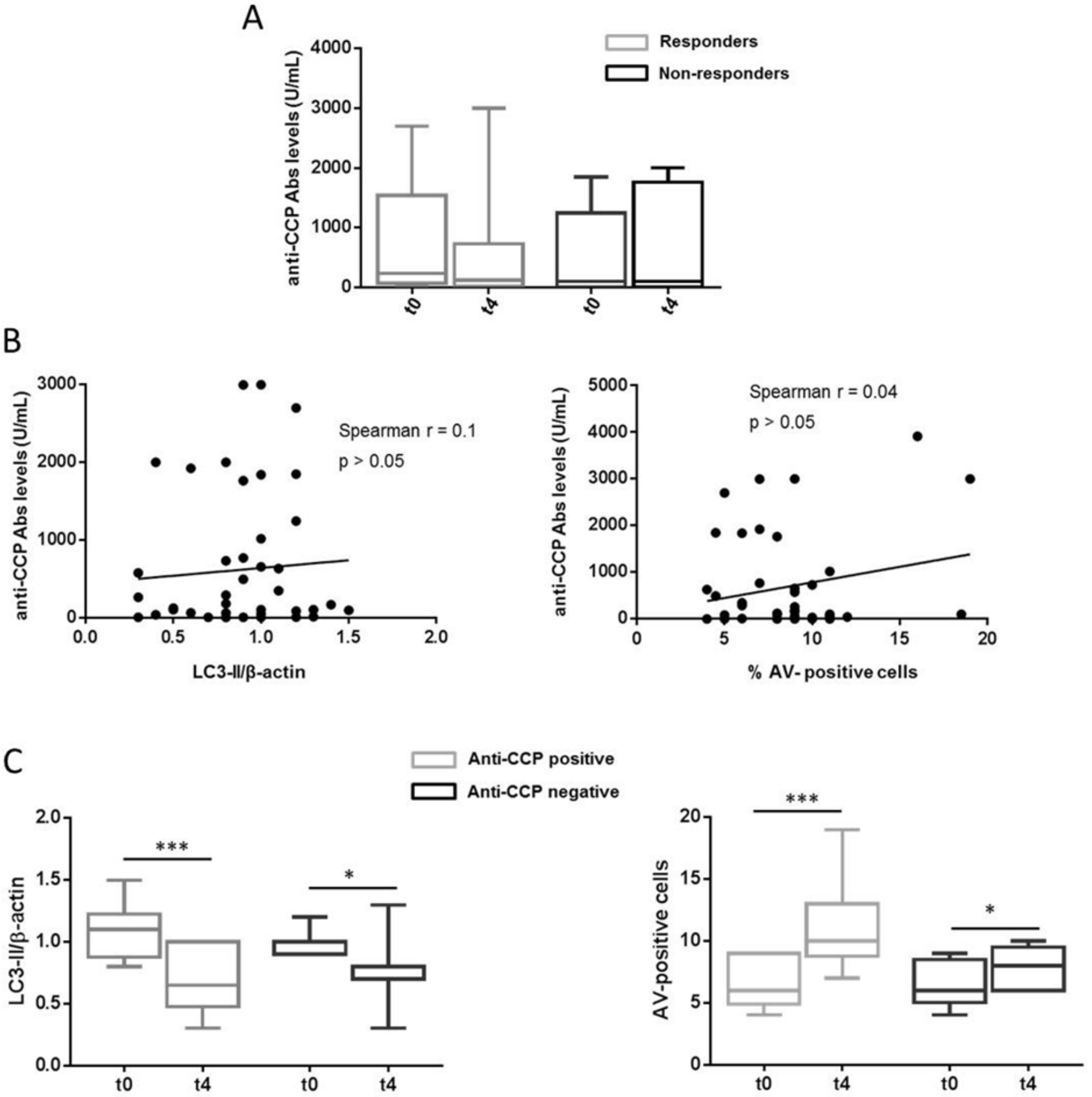

Supplementary Figure 2. Relation between autophagy and citrullination in RA patients.

A. Levels of anti-cyclic citrullinated peptide antibodies (anti-CCP Abs) in patients with RA before and after treatment with anti-TNF drugs. Values are expressed as means ± sd. B. Correlation between anti-CCP antibodies levels (U/ml) and spontaneous autophagy (expressed as LC3-II) and apoptosis (expressed as percentage of Annexin V-positive cells). C. Changes in autophagy and apoptosis before and after treatment with anti-TNF drugs in relation to anti-CCP in RA responding patients.

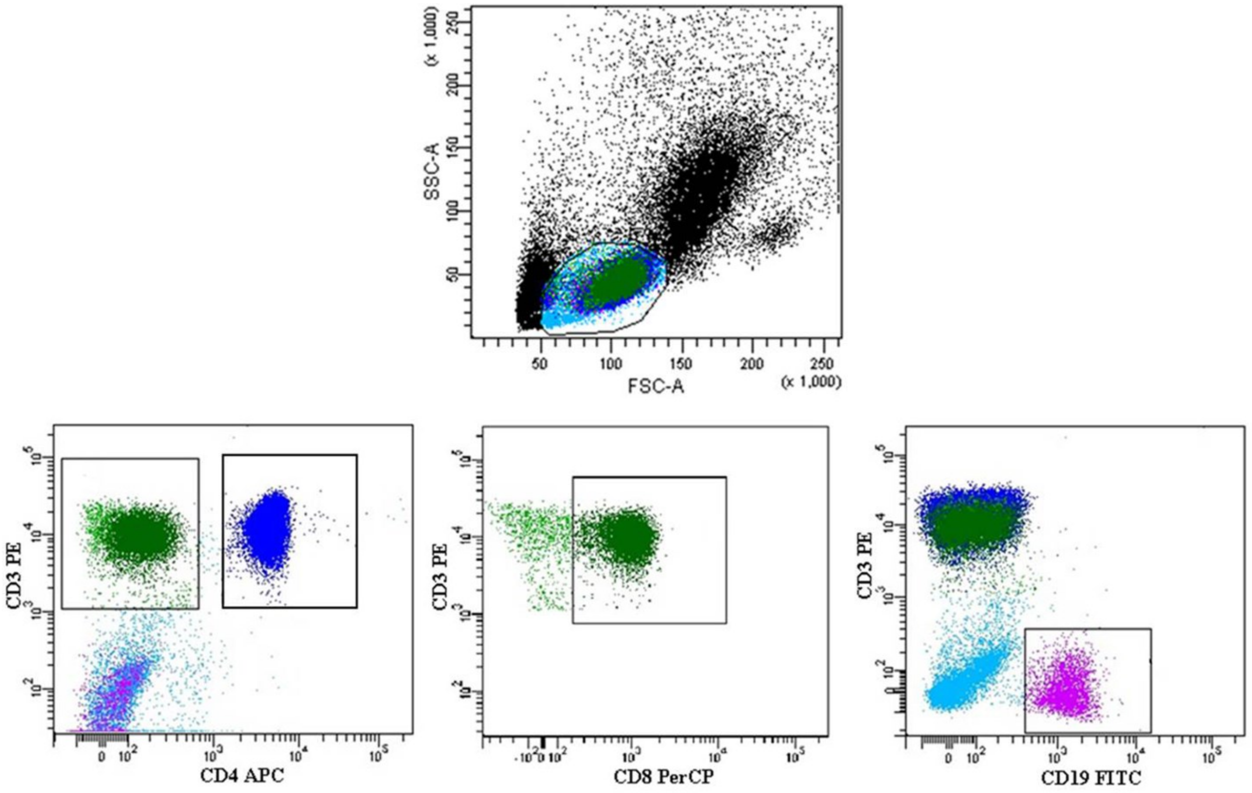

Supplementary Figure 3. Flow cytometry gate strategy for purification of CD4+, CD8+ T lymphocytes and B lymphocytes from patients with RA.

Allophycocyanin = APC; Peridinin chlorophyll protein = PerCP; Phycoerythrin = PE; Fluorescein isothiocyanate = FITC.

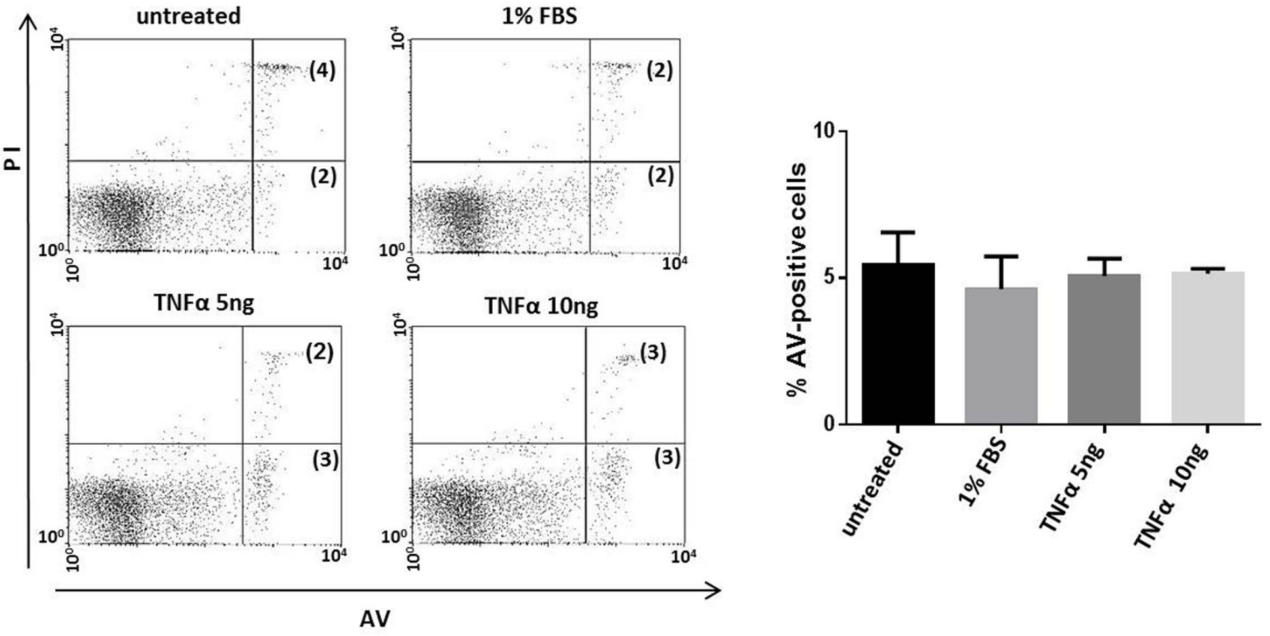

Supplementary Figure 4. Effect of TNFα on apoptosis. Flow cytometry analysis of apoptosis in PBMCs after treatment with TNFα. Apoptosis is expressed as percentage of AV-positive cells. Representative dot plots (PI on y axis vs. AV on x axis), chosen as representative of five experiments, are also shown.

Characteristic	Value
**Demographic parameters**	
**Sex, F/M**	**6/2**
Age, mean (SD), years	51 (13)
**Disease duration, mean (SD), years**	**10 (9.3)**
**Laboratory parameters**	
**ESR, mean (mm/h) (SD)**	**21 (12)**
CRP, mean (mg/dL) (SD)	0.85 (1.1)
**RF positivity, n (%)**	**5 (62.5)**
ACPA positivity, n (%)	5 (62.5)
**Disease activity**	
TJ n, mean (SD)	6.2 (5.1)
SJ n, mean (SD)	4.1 (5.9)
CDAI, mean (SD)	22.3 (15.9)
DAS28, mean (SD)	4.6 (1.9)
**Therapy**	
**Etanercept, n (%)**	**6 (75)**
Adalimumab, n (%)	2 (25)
**Concurrent MTX, n (%)**	**6 (75)**

Supplementary Table 1. Clinical, demographic and serological characteristics of patients with RA enrolled for sorting experiments (n = 8).
